# Social Support, Health Literacy and Depressive Symptoms among Medical Students: An Analysis of Mediating Effects

**DOI:** 10.3390/ijerph18020633

**Published:** 2021-01-13

**Authors:** Yaqin Zhong, Elizabeth Schroeder, Yuexia Gao, Xiaojun Guo, Yuanyuan Gu

**Affiliations:** 1 School of Public Health, Nantong University, Nantong 210029, China; yqzhong@ntu.edu.cn (Y.Z.); gaoyuexia1103@163.com (Y.G.); 2 Macquarie University Centre for the Health Economy, Macquarie Business School, Macquarie University, Macquarie Park, NSW 2109, Australia; liz.schroeder@mq.edu.au; 3 Department of Health Systems and Populations, Macquarie University, Macquarie Park, NSW 2109, Australia; 4 School of Sciences, Nantong University, Nantong 210029, China; guoxj159@163.com

**Keywords:** health literacy, social support, depressive symptoms, mediation effects, medical students

## Abstract

Depressive symptoms are prevalent in university students and may impair their social, educational, and economic transition into adulthood. Identifying the factors that determine depressive symptoms is crucial for the design of effective policy interventions. This study aims to examine the associations between health literacy and depressive symptoms among medical students, and to evaluate the effect of different types of social support as a potential mediator. A cross-sectional survey of medical students was conducted through convenience sampling in East China. Associations between variables were explored using OLS and the mediation effect was estimated using the Karlson, Holm and Breen method. A total of 746 valid questionnaires were collected. The prevalence of depressive symptoms among the sample was 32.4%. Higher health literacy levels and social supports were significantly associated with lower levels of depressive symptoms. Social support partially mediated the association between health literacy and depressive symptoms, accounting for a 54.03% of the total effect size. These findings suggest that interventions for medical student mental wellbeing could improve health literacy. Whilst family support reflects greatest impact, Universities can also lead and innovate novel interventions for this critical stage of life. Future research can extend this study by exploring the dynamic interactions between health literacy, depressive symptoms, and other sources of social support. Comparisons of these findings across the different regions of China and in other university subject disciplines are also warranted.

## 1. Introduction

Depressive symptoms, which are typically characterized by hopelessness, sadness and/or anxiety, are prevalent among university students globally [1,2,3]. Systematic reviews suggest a prevalence of approximately 33% for depressive symptoms across all university students [4] and slightly less at 27.2% for medical students [5] though the overall prevalence is increasing [6]. In China, this pattern is slightly reversed, with an estimated prevalence of 23.8% among all students [7] but heightened to 29.0% in medical students [8]. 

In general, when averaged across the lifespan, students tend to have a higher prevalence of depressive symptoms than the general population [7,9]. Whilst the context is not specifically explored here, commencement of university or college life reflects numerous, sudden, complex and multi-component life changes, which may exacerbate student vulnerability, requiring deeper emotional resilience. Pressures may also include academic expectations, interpersonal relationships, unfamiliar environments and financial stress. Depressive symptoms are detrimental to student quality of life and the evidence suggests that the students’ social functioning and academic performance may also be affected [4]. There may be episodic trends across the lifespan during times of specific vulnerability, and adolescent depression heightens the likelihood of depression in adulthood, which has also been linked to suicidal ideation [10]. The detrimental effect of depressive symptoms is likely to be more damaging for students with pre-existing mental health conditions. The overlap of the transition to emerging adulthood, an unfamiliar university environment and the loss of previous social supports can be particularly challenging, given their vulnerabilities and potential comorbidities. 

As in many other countries, medical students in China experience considerable academic pressure, and so this research investigates a cohort of students with exposure to high stress. [8]. In addition, they experienced the isolating and clinically taxing effects of the COVID-19 pandemic, exacerbating negative effects on their mental health, and compounding the well-documented stress of their professional commitments, responsibilities and working practices [11,12]. This cohort therefore provides a unique perspective and data towards analysis of the interactions of determining factors for depressive symptoms, and to inform effective policy interventions. 

Reviews of published literature suggest that many factors may interact, often aligned to the social determinants of health [13]. These include demographic characteristics (e.g., gender, age, birthplace) [14], social interaction (social support, social network) [15,16], health literacy [17,18], health status [16] and family circumstances (e.g., parents’ education, family economic status) [9,16].

In this study, we investigate the association between health literacy and depressive symptoms among medical students, using the econometric approaches of OLS regressions with mediation effects for social support. This allows us to estimate factors in the association between health literacy and depressive symptoms. To our knowledge this is the first paper exploring this topic.

### 1.1. Health Literacy and Depressive Symptoms

Health literacy (HL) is described as the “cognitive and social skills which determine the motivation and ability of individuals to gain access to, understand and use information in ways which promote and maintain good health” [19]. Health literacy tends to be under-valued, but the evidence suggests it is an independent and direct predictor of health outcomes [20,21,22] and people with lower levels of health literacy are more likely to report worse health-related behaviors, poorer use of health care services and poorer health status [23]. In some countries, such as China and Korea, the promotion of health literacy has therefore been engaged as a key strategy to reduce health inequities [20,21,22].

There is a paucity of evidence for the relationship between health literacy (HL) and depression, in particular regarding measures of association, and the findings are mixed. A study conducted with 489 HIV-seropositive adults showed that individuals with higher HL reported more depressive symptoms [24]. Research conducted with elderly Latino and Korean populations, found lower HL was associated with higher levels of depressive symptoms. A study conducted among 585 Korean adults found that health literacy plays an important role in preventing depression after controlling for sociodemographic and health-related characteristics [22]. Another study conducted among 3260 elderly Medicare enrollees in the United States found that the associations between HL and depressive symptoms disappeared when controlling for confounding, including chronic disease conditions and self-rated health [25].

The variability of prior research raises a concern about the robustness of the relationship between HL and depression. This may be caused by study heterogeneity, such as controlling for different confounding factors or narrowed to select age groups. Some studies, especially those with a low response rate, may also suffer from self-selection bias. Our study aims to explore the relationship between HL and depression in students, leading to an empirical test of the first hypothesis:

**Hypothesis** **1.** *Health literacy is negatively associated with depressive symptoms.*

### 1.2. Mediation—Social Support and Depressive Symptoms

Social support is typically defined as “the existence or availability of people on whom we can rely, and people who let us know that they care about, value, and love us” [26]. Social support comprises ‘interpersonal resources that are accessed and mobilized when dealing with life challenges’ [27]. It broadly encompasses emotional and material supports and can be as insubstantial as information or knowledge sharing between family, friends, and significant others [15]. Previous studies have identified a significantly protective relationship (here negative association) between social support and depressive symptoms [15,16,27,28]. For example, Zhang found a protective effect of social support in the relationship between stress and depression in high school students [15]. A study that examined the relationship between perfectionism and depression among 426 Chinese university students found that perceived social support was protective for depression [29]. In another, social support served as a mediator between stress and psychological symptoms [30]. Similarly, for caregivers of disabled people; spousal and family support was positively correlated with wellbeing [27]. This leads to a second hypothesis:

**Hypothesis** **2.** *Social support is negatively associated with depressive symptoms.*

### 1.3. Social Support as a Mediator between Health Literacy and Depression

Studies have explored the relationships between health literacy, social support and depression. In the United States in Medicare enrollees, Gazmararian identified a protective relationship for social support between HL and depressive symptoms [25]. Another identified that among racially and ethnically diverse smokers of lower SES and lower HL, lower perceived support and higher rates of depressive symptoms could be predicted [31]. These findings suggest that social support might function as a mediator through which HL impacts depression. The Social Determinants of Health frameworks capture these interactions comprehensively, but less is known when applying the lens to health literacy. People with lower HL have reported feelings of shame in their difficulty in understanding health information, also heightened perceptions of lower social support, which may, in turn, cause helplessness, isolation and raised levels of depression [31]. Seligman’s theory of learned helplessness suggests that depression arises from a perceived lack of autonomy or control, impacting those with poor HL and support more, and possibly being cyclical in nature [32].

Medical students do not tend to be overwhelmingly represented as low SES, and tend to have high rates of HL, educational attainment and articulation. However, as mentioned, their uniquely pressurized and competitive environments, vulnerability in life transitions, and perceived lack of autonomy in work-life balance, leads them to be an important cohort to research. More broadly, little is known about the protective effects of social support for medical students. Here we propose the third hypothesis:

**Hypothesis** **3.** *Social support mediates the association between health literacy and depressive symptoms.*

## 2. Methods

### 2.1. Sampling and Data Collection

From April to May 2020, a cross-sectional survey of medical students was conducted at three university campuses in East China (*n* = 746, campus Seyuan = 429, campus Qixiu = 172 and campus Zhongxiu = 145). All medical students physically located on three campuses were included. 

At the time of the survey, a strict COVID-19 lock-down was recently concluded, students had returned to campus or were studying online at home. Most first-year students studied online from home, exempt from laboratory research and the use of campus facilities. It became too challenging to reach them, thus, first year students were excluded.

A convenience sampling approach to data collection was used. Students were informed of the purpose, confidentiality, and voluntary nature of participation prior to consent. The target sample size was 700–800, with 24 of 150 advisor-led classes (30–40 students per class) selected at random, providing a sample size of 836 students. 746 questionnaires were completed and cleaned, suggesting a response rate of 89%. The questionnaires included validated questions and measures of individual characteristics, family circumstances, health literacy, social support and depressive symptoms.

### 2.2. Measurements

#### 2.2.1. Depressive Symptoms

Depressive symptoms were the primary outcome, and were assessed from scoring obtained from the Self-rating Depression Scale (SDS) [33]. The Chinese version of SDS, used here, has good reliability and validity [34]. The SDS includes 20 items about the frequency of symptoms experienced. Each item is assessed on a 4-point Likert-type scale (1 = no or little time; 4 = most or all the time). Of 20 items, ten are worded positively and ten negatively. Ten positive items are reverse-scored, and the total scores range between 20 and 80, with higher scores indicating more severe depressive symptoms. The lowest threshold for depressive symptoms is a score of 53. 

#### 2.2.2. Health Literacy

The Chinese eight-item Health Literacy Assessment Tool (c-HALT-8) [20] was used to measure health literacy. The HALT-8 was developed in Switzerland in 2014 [35], and was translated from English to Chinese (c-HALT-8) [20]. It has shown good evidence of construct validity and reliability for young adults in China [20]. It consists of eight items to measure the capability to assess, understand, communicate and evaluate health information in everyday life. It covers three domains; functional, interactive and critical health literacy, thus enabling a more comprehensive examination of the construct [35]. Scores range from 0 to 37, and higher scores indicate higher levels of health literacy.

#### 2.2.3. Social Support

Social support was measured by the Multidimensional Scale of Perceived Social Support (MSPSS) [36], with the Chinese version used in this study [37]. The MSPSS consists of twelve questions that assess support from family, friends, and significant others, accommodating for the respondents’ age, social status and cultural context. Teachers and classmates were included and variable reliability was checked using Cronbach alpha scoring (0.969). Respondents assessed each item on a 7-point Likert type scale and total scores ranged between 12 and 84; higher scores indicate stronger social support.

#### 2.2.4. Other Variables

Our previous studies reported potential demographic or confounding variables [9,17,21,23]. Participant characteristics include gender, academic performance, and self-rated health (SRH). Family characteristics include birthplace, socioeconomic status and parent’s education. Parent’s education was recoded to binary categories: [high school or less (reference level); tertiary]. Perceptions of academic performance were measured by the question “how does your academic performance compare with your classmates” [poor (reference category); fair; good]. 

### 2.3. Statistical Analyses

The first two hypotheses were tested using an OLS regression and the third with OLS and then formally tested with mediation. The scores of health literacy, social support and depressive symptoms were centered before analyses.

Four OLS regression models were estimated. In model 1, health literacy and depressive symptoms were analysed, and the dependent variable was depressive symptoms (Hypothesis 1). In model 2, health literacy and social support were analysed, and the dependent variable was social support. Model 3 analysed social support and depressive symptoms, and the dependent variable was depressive symptoms (Hypothesis 2). In model 4, the dependent variable was depressive symptoms with both health literacy and social support included (for an analysis of direct effects on the outcome variable). These four models offered insights on how factors interact, and their magnitude. In all models, the confounding effects of demographic and family characteristics were controlled. To investigate whether different sources of social support were associated with depressive symptoms, OLS regression was also conducted controlling for potential covariates.

The Karlson, Holm and Breen (KHB) method was used to assess whether social support had a mediating effect on health literacy and depressive symptoms [38]. The total effect was deconstructed into direct effects (health literacy on depressive symptoms when controlling for social support) and indirect effects (health literacy on depressive symptoms through the mediating variable of social support). The proportion is calculated as the indirect effect divided by the total effect. All statistical analyses were conducted using STATA 14.0. A standard approach to inference was applied with a *p*-value less than 0.05 considered statistically significant.

## 3. Results

### 3.1. Descriptive Statistics

Sociodemographic characteristics are reported in Table 1. There were more females (67.3%) than males (32.7%). The age range of the students was from 18 to 24 years. Place of birth was 29.8%, 33.9% and 36.3% for city, town and county, respectively. Parental education attainment between groups was similar; education below high school for fathers was 40.3% and 48.4% for mother’s education. Participant academic record self-reports were poor (22.6%), fair (47.5%) and good (26.5%). Baseline scores for SRH was 43.7% (good) and 5.6% (poor). The average score for health literacy was 25.69, surprisingly lower than that of Chinese secondary school students [20]. Average baseline scores were 62.71 (social support, range 12 to 84) and 47.86 (depressive symptoms, range 25 to 84). The Cronbach’s α for SDS in our study was 0.858, which meant good reliability.

### 3.2. Effects of Health Literacy and Social Support on Depressive Symptoms

The estimates of models 1–4 are documented in Table 2. In model 1, heath literacy reflects a significant negative coefficient, which suggests that health literacy is negatively associated with depressive symptoms. Students who rated their family economic status as “poor” and rated their health as “fair” (as opposed to ‘good”) were associated with more depressive symptoms. In model 2, higher literacy was associated with more social support. Females reported more social support compared to males and students who had good academic performance also reported more social support. In model 3, social support was negatively associated with depressive symptoms. In model 4, the health literacy and social support were both negatively associated with depressive symptoms. Model 4 explained 30.4% of the variance of depressive symptoms. Separately, when all covariates were controlled good academic performance was associated with reduced depressive symptoms.

Table 3 reports an association between health literacy, three sources of social support (family, friends, teachers) and depressive symptoms. While health literacy was negatively associated with depressive symptoms, only social support from family was negative and significant. In other words, students with more support from family had lower level of depressive symptoms and of the sources of support, family had the most impact.

### 3.3. The Mediation Effect of Social Support

**Figure 1:**
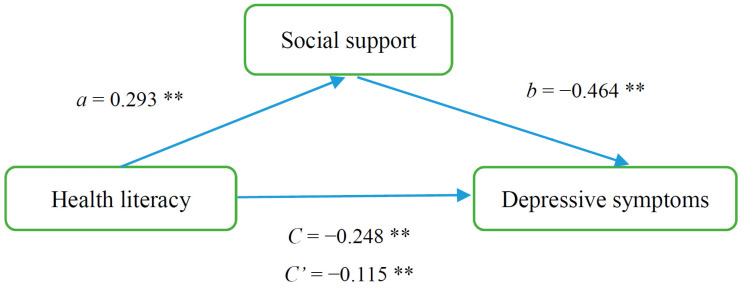
The mediating effect of social support on the relationship between health literacy and depressive symptom. ** *p* < 0.01.

The results of the model are shown diagrammatically in Figure 1. Broadly, social support had a mediation effect between health literacy and depressive symptoms. Paths *a, b* and *c* represent standardised coefficients between the paths. Path *c* presents the link between health literacy and depressive symptoms, *a* presents the link between health literacy and social support; *b* shows social support and depressive symptoms; and *c’* shows the effect of health literacy on depressive symptoms incorporating mediating social support.

Table 4 shows the significant test of the mediating pathways. Our results indicated that the association between health literacy and depressive symptom was partially mediated by social support (95% CI: 0.840–0.904, with an indirect effect, (*ab* = *c* − *c*′) of social support of 0.134, which accounted for 54.03% of the total effect.

## 4. Discussion

College students with lower HL reported more depressive symptoms. This finding is consistent with previous studies [21,22]; lower heath literacy may be an independent risk factor for depressive symptoms. Social support was negatively associated with depressive symptoms after potential covariates were controlled, proving protective as a factor against depression. Social support is important and accounted for 54.03% of the total effect between health literacy and depressive symptoms. Within the different sources of social support, support from family proved significant, suggesting it represents the most cogent mediator role or perhaps that other potential sources of social support have less impact and could be enhanced. This may merit more research and bigger study samples, as the evidence is mixed. All three sources of social support were significant moderators of depressive symptoms for adolescents in suburban communities in USA [39]. However, findings similar to ours were identified in a study of African American youth in low-income urban schools [40]. 

The prevalence of depressive symptoms was 32.4%, which was higher than in previous studies. A meta-analysis analysed depressive symptoms in Chinese medical students with a prevalence of 29.0% [8]. There is a chance that the timing of data capture during the COVID 19 pandemic impacted our findings; and seemed to impact all scores negatively. It would seem that social isolation, social and academic disruption and uncertainty about their future had a significant impact on all students. 

The average score of health literacy was 25.70 (±4.52), slightly lower than published scores for Chinese secondary school students 26.37(±5.89), but the difference is likely not significant [20]. The study published data from scholars in Beijing, a city with more sophisticated health promotion, and with a cohort likely to have higher levels of health literacy and healthier behaviors. 

We also found that students with good academic performance had lower levels of depressive symptoms. This was inconclusive of cause/effect measured in other studies [41,42], though depression is often associated with feelings of hopelessness and a lack of motivation and is predictably associated with a poor academic performance [43].

## 5. Implications

Whilst this research is based on a convenient sample, our study touches on policy implications that can be explored extensively in future research. The identification of mechanisms that could enhance health literacy as a strategic intervention may help to prevent and alleviate depressive symptoms. Currently, students reflect a strong protective effect from family support, though the evidence identifies more areas where additional support, or enhancements to health literacy can prove valuable. Universities can play a critical role in providing early health-promoting interventions for their students, with an emphasis on health literacy and mental and physical health self-management. Curriculums that incorporate mental health education could be offered to medical college students early in their learning journey. Resilience training programs, legal supports, financial advice, and other aids to strengthen student wellbeing can provide value, including the supply of counsellors, tutors and other forms of pastoral oversight. The implementation of strategies derived from the Australian government and university-led student experience surveys (SES-QILT) can also provide exemplars. The surveys, whilst capturing domains broader than health, identify scaffolding that can support student transitions into adult learning trajectories through skills development, learner engagement, teaching quality, student support, and the adequate provision of learning resources. Initiatives to improve these domains can realign student academic engagement and achievement, and well-being.

In addition, the study captures the moderating effects of social support on both HL and depression. Students with lower HL may benefit from eliciting support from their families and valuing the involvement of a supportive family in any care or treatment pathways, whether local or abroad. More so, harnessing social support opportunities in Universities for mental wellbeing is under-researched. High-level facilitation of social support for students (student or teacher-led networking, tutorial group activities) and other interactive engagement is increasingly diminished as instruction moves online—but the benefits of face-to-face interaction for student wellbeing might outweigh the costs. Currently unexplored are clinical treatment pathways for students with depressive symptoms, in terms of help-seeking, offer, uptake and utilisation. Positive psychology interventions (PPI) have been developed to increase positive affectivity. Previous studies focused on the efficacy of PPI showed it is efficacious in reducing depressive symptoms as well as increasing well-being [44]. 

## 6. Limitations

Our study has several limitations. First, the study sample was self-selected and limited to medical college students from one university with data collected during COVID-19 conditions. It is therefore not representative of the whole medical college student population in China. These factors may have impacted the results, and we did identify depression rates that were higher than global averages, though only slightly. However, baseline demographics and econometric modelling results did show alignment to other research. Extending the research to different regions across China and to other university subject disciplines should be considered. Second, the self-Rating Depression Scale was used as a depression screen tool instead of a clinical diagnosis from psychiatrists. Self-report can over-estimate the baseline depressive symptoms and overstate the benefits of interventions. Irrespective, the instrument is a validated tool for depression identification and will have internal validity with other studies using the same instrument. Third, the study is cross-sectional and does not allow for causal inferences nor analyses of change from the trend over time. Longitudinal, registry or panel datasets capturing data could underpin a more rigorous and robust approach to estimating mediating and moderating effects and cause and consequence. Finally, we used c-HALT-8 as the health literacy measurement tool, which was developed for young people, not for medical students specifically.

## 7. Conclusions

This study represents the first effort to examine the association between health literacy and depressive symptoms among medical college students. Our results confirmed a significant negative association between them and the mediating role of social support. Future studies should replicate and extend this study to explore the dynamic interactions of health literacy and self-management, depressive symptoms and different sources of social support.

## Figures and Tables

**Table 1:** Characteristics of medical college students.

Variables	*n* (%)	Mean ± SD
Gender		
Male	244 (32.7)	
Female	502 (67.3)	
Academic record		
Poor	194 (26.0)	
Fair	354 (47.5)	
Good	198 (26.5)	
Birthplace		
City	222 (29.8)	
Town	253 (33.9)	
County	271 (36.3)	
Father’s education		
Below high school	301 (40.3)	
High school and above	445 (59.7)	
Mother’s education		
Below high school	361 (48.4)	
High school and above	385 (51.6)	
Family economic status		
Good	99 (13.3)	
Fair	546 (73.2)	
Poor	101 (13.5)	
Self-rated health		
Good	326 (43.7)	
Fair	378 (50.7)	
Poor	42 (5.6)	
Health literacy		25.698 ± 4.521
Social support		62.719 ± 14.241
Depressive symptoms		47.867 ± 11.105
Yes	242 (32.4)	
No	504 (67.6)	

**Table 2:** Association between health literacy, social support and depressive symptom.

Variables	Model 1 ^a^		Model 2 ^b^		Model 3 ^a^		Model 4 ^a^	
	*β*	SE	*β*	SE	*β*	SE	*β*	SE
Health literacy	−0.248 **	0.035	0.294 **	0.035			−0.115 **	0.033
Social support					−0.500**	0.033	−0.464 **	0.034
Gender ^1^								
Female	−0.086	0.076	0.233 **	0.073	0.060	0.068	0.034	0.068
Academic performance ^2^								
Fair	−0.121	0.086	0.086	0.084	−0.106	0.077	−0.087	0.077
Good	−0.276	0.098	0.197 *	0.095	−0.214 *	0.088	−0.192 *	0.088
Birthplace ^3^								
Town	−0.092	0.097	0.108	0.094	−0.049	0.087	−0.040	0.087
County	−0.064	0.089	0.083	0.086	−0.041	0.080	−0.040	0.080
Father’s education ^4^								
Higher school and above	0.020	0.091	0.124	0.088	0.088	0.081	0.080	0.081
Mother’s education								
Higher school and above	0.198 *	0.089	−0.218 *	0.086	0.073	0.079	0.098	0.079
Family economic status ^5^								
Fair	−0.091	0.147	0.386	0.144 **	0.102	0.134	0.105	0.133
Poor	−0.374 **	0.108	0.488	0.106 **	−0.133	0.099	−0.130	0.099
Self-rated health ^6^								
Fair	−0.466 **	0.161	0.445 **	0.157	−0.281	0.146	−0.264	0.145
Poor	−0.207	0.158	0.196	0.154	−0.118	0.142	−0.123	0.141
Adj R^2^	12.7%		17.7%		29.3%		30.4%	

^1^: reference = male; ^2^: reference = poor; ^3^: reference = city; ^4^: reference = below high school; ^5^: reference = good; ^6^: reference = good; ^a^: Dependent variable = depressive symptoms; ^b^: Dependent variable = social support; * *p* < 0.05, ** *p* < 0.01.

**Table 3:** Association between health literacy, three sources of social support and depressive symptom.

Variables	*β*	SE
Health literacy	−0.121 **	0.033
Social support from teachers and classmates	−0.124	0.069
Social support from family	−0.287 **	0.056
Social support from friends	−0.084	0.067
Gender ^1^: Female	0.027	0.068
Academic performance ^2^		
Fair	−0.073	0.077
Good	−0.174 *	0.088
Birthplace ^3^		
Town	−0.048	0.087
County	−0.059	0.080
Father’s education ^4^: Higher school and above	0.072	0.081
Mother’s education ^4^: Higher school and above	0.111	0.079
Family economic status ^5^		
Fair	0.133	0.134
Poor	−0.118	0.099
Self-rated health ^6^		
Fair	−0.259	0.145
Poor	−0.102	0.142
Adj R^2^	30.6%	

^1^: reference = male; ^2^: reference = poor; ^3^: reference = city; ^4^: reference = below high school; ^5^: reference = good; ^6^: reference = good; * *p* < 0.05, ** *p* < 0.01.

**Table 4:** Models of the mediating role of social support in the relationship between health literacy and depressive symptoms.

Effect	*β*	SE	Z	*p*	OR (95% CI)
Total effect (*c*)	−0.248	0.025	−7.97	<0.001	0.776 (0.730–0.826)
Direct effect (*c*′)	−0.115	0.030	−3.47	0.001	0.891 (0.835–0.951)
Indirect effect (*ab* = *c* − *c*′)	−0.134	0.016	−7.31	<0.001	0.872 (0.840–0.904)

## Data Availability

The data generated and analysed in the current study are not publicly available. Please contact Yaqin Zhong for the data.
